# Increased Risk of Severe Gastric Symptoms by Virulence Factors *vacAs1c*, *alpA*, *babA2*, and *hopZ* in *Helicobacter pylori* Infection

**DOI:** 10.4014/jmb.2101.01023

**Published:** 2021-02-08

**Authors:** Dong-Hae Lee, Jong-Hun Ha, Jeong-Ih Shin, Kyu-Min Kim, Jeong-gyu Choi, Seorin Park, Jin-Sik Park, Ji-Hyeun Seo, Ji-Shook Park, Min-Kyoung Shin, Seung-Chul Baik, Woo-Kon Lee, Hee-Shang Youn, Myung-Je Cho, Hyung-Lyun Kang, Myunghwan Jung

**Affiliations:** 1 Department of Microbiology, College of Medicine, Gyeongsang National University, Jinju 52727, Republic of Korea; 2 BK21 Center for Human Resource Development in the Bio-Health Industry, Department of Convergence Medical Science, Gyeongsang National University, Jinju 52727, Republic of Korea; 3 Department of Pediatrics, College of Medicine, Gyeongsang National University, Jinju 52727, Republic of Korea; 4 Institute of Health Science, Gyeongsang National University, Jinju 52727, Republic of Korea; 5 Research Institute of Life Science, Gyeongsang National University, Jinju 52828, Republic of Korea

**Keywords:** *Helicobacter pylori*, *vacAs1c*, *alpA*, *babA2*, *hopZ*, gastric diseases

## Abstract

Two virulence factors of *Helicobacter pylori*, *cagA* and *vacA*, have been known to play a role in the development of severe gastric symptoms. However, they are not always associated with peptic ulcer or gastric cancer. To predict the disease outcome more accurately, it is necessary to understand the risk of severe symptoms linked to other virulence factors. Several other virulence factors of *H. pylori* have also been reported to be associated with disease outcomes, although there are many controversial descriptions. *H. pylori* isolates from Koreans may be useful in evaluating the relevance of other virulence factors to clinical symptoms of gastric diseases because the majority of Koreans are infected by toxigenic strains of *H. pylori* bearing *cagA* and *vacA*. In this study, a total of 116 *H. pylori* strains from Korean patients with chronic gastritis, peptic ulcers, and gastric cancers were genotyped. The presence of virulence factors *vacAs1c*, *alpA*, *babA2*, *hopZ*, and the extremely strong vacuolating toxin was found to contribute significantly to the development of severe gastric symptoms. The genotype combination *vacAs1c*/*alpA*/*babA2* was the most predictable determinant for the development of severe symptoms, and the presence of *babA2* was found to be the most critical factor. This study provides important information on the virulence factors that contribute to the development of severe gastric symptoms and will assist in predicting clinical disease outcomes due to *H. pylori* infection.

## Introduction

*Helicobacter pylori* is a gram-negative, spiral-shaped capnophilic bacterium [1] which is closely associated with the epithelial surface or the surface mucus of gastric mucosa [2]. *H. pylori* is the causative agent of chronic gastritis and peptic ulcer, and prolonged carriage is known to be a significant risk factor for the development of gastric cancer [3-7]. Therefore, *H. pylori* was defined as a Class 1 carcinogen by the World Health Organization (WHO) in 1994, and estimates indicate that more than half of the global population is infected by *H. pylori* [7]. In 2020, the International Agency for Research on Cancer reported that *H. pylori* was responsible for the most infectious pathogen-related carcinogenesis in 2018 (https://gco.iarc.fr/causes/infections). In addition, most Koreans are carriers of *H. pylori* by early childhood [8], which is believed to be a major reason for the high prevalence of gastric disorders including gastric cancer in Korea [9].

The incidence of gastric cancer differs geographically [10], and East Asian countries have the highest incidence rates of this disease worldwide [11]. This regional discrepancy can be partly explained by different prevalence rates of *H. pylori* infection [11], which in East Asian countries are reported to be higher than those in Western countries [8]. However, the incidence of gastric cancer in countries located in West South Asia is similar to that in Western countries, even though *H. pylori* infection is more prevalent in West South Asia than in Western countries [11]. These studies suggest that other factors in addition to infection could play a role in the pathogenesis of *H. pylori* and the development of pestilential gastric symptoms.

*H. pylori* is a highly heterogeneous bacterium [12], and various virulence factors have been identified in isolates [5, 13]. The *cagA* and *vacA* genes have been extensively studied as virulence markers of *H. pylori* strains, and toxigenic strains of *H. pylori* carrying *cagA* and *vacA* are closely associated with the development of gastric diseases such as peptic ulcer and gastric cancer [2, 10, 11, 14, 15]. More *cagA*-positive strains of *H. pylori* have been recovered from East Asian populations than from Western populations [16], and more *vacA*-positive strains have been recovered from patients with peptic ulcer than from patients without peptic ulcer [17, 18]. These studies on *cagA* and *vacA* suggest that these virulence factors play an important role in the development of clinical entities of gastric diseases. However, although infections of *H. pylori* bearing *cagA* and *vacA* cause chronic gastritis in many cases, they are not always associated with peptic ulcer or gastric cancer [3]. Only approximately 10–15% of infected individuals were reported to develop peptic ulcer, gastric carcinoma, and mucosa associated lymphoid tissue (MALT) lymphoma [6, 19]. Similarly, in Korea, even though the majority of people become infected early in life with toxigenic *H. pylori* strains bearing *cagA* and *vacA* [20-22], only a small proportion of infected Koreans complain of severe gastric maladies [21-23]. These studies suggest that additional virulence factors of *H. pylori* are involved in disease outcome.

In addition to CagA and VacA, other virulence factors, including adherence proteins, endonuclease restriction/modification systems, and *vir* homologs have been explored to identify their relevance to the development of different outcomes of gastric diseases. AlpA/B [17, 24, 25], BabA2 [26-28], HopZ [29], OipA [30, 31], SabA [3, 5, 32], HrgA [14], HpyIII [14], IceA [33], and *vir* homologues [22] have been reported to be partly associated with disease outcomes, although there are many controversial descriptions. Therefore, it is necessary to evaluate which of these virulence factors, in addition to CagA and VacA, critically contribute to the development of severe symptoms of gastric diseases.

Most *H. pylori* isolates from East Asian countries, including Korea, contain both an East Asian repeat pattern in the 3′-region of the *cagA* gene and the toxigenic *vacA* genotype, irrespective of the presentation of gastric symptoms [11, 21, 23, 34]. Therefore, *H. pylori* isolates recovered from the Korean population are of value in evaluating the relevance of other virulence factors in the development of clinical outcomes in addition to *cagA* and *vacA*. Here, we assessed Korean isolates of *H. pylori* by genotyping for different virulence factors including vacuolating toxin, adherence proteins, endonuclease restriction/modification systems, and *vir* homologues. In addition, we analyzed the relevance of these genes to the clinical symptoms of gastric disorders and investigated the polymorphisms of *cagA* and vacuolating toxin activities.

## Materials and Methods

### *H. pylori* Isolates

*H. pylori* strains were isolated at Gyeongsang National University Hospital (Korea), Kosin University Gospel Hospital (Korea), and St. Carollo Hospital (Korea) from 1987 to 2005, as previously described [35-37]. Briefly, biopsy specimens were inoculated by smearing onto Mueller-Hinton agar (Merck, USA) plates containing 10%bovine serum (Gibco, USA), vancomycin (Merck, 10 μg/ml), nalidixic acid (Merck, 25 μg/ml), and amphotericin B (Merck, 1 μg/ml). The plates were incubated at 37°C under 10% CO_2_ and 100% humidity for 7 days. The bacteria were identified as *H. pylori* on the basis of their morphology, urease test, and 16S rRNA PCR. To amplify the 16SrRNA gene of *H. pylori*, primers of PV31 (5'-CGGCCCAGACTCCTACGGG-3') and PV32 (5'-TTACCGCGG CTGCTGGCAC-3') were designed based on the previous publication [38]. The PCR amplification was performed with AccuPower PCR PreMix (Cat. No. K-2016, Bioneer, Korea) containing 1 unit of *Top* DNA polymerase, 250 μM dNTPs, 10 mM Tris-HCl (pH 9.0), 30 mM KCl, 1.5 mM MgCl2, and 10 pmol of the primers. The PCR condition was as follows: pre-denaturation at 94°C for 5 min; followed by denaturation at 94°C for 30 sec, annealing at 55°C for 30 sec, extension at 72°C for 3 min (35 cycles), and a final extension at 72°C for 7 min. PCR-amplified products (180 bp) were detected by agarose gel electrophoresis to identify *H. pylori*.

All isolates were deposited at the former *H. pylori* Korean Type Culture Collection (HpKTCC; College of Medicine, Gyeongsang National University, Korea; 2006–2015) with anonymized clinical information. Among the deposited strains, *H. pylori* strains recovered from patients with chronic gastritis, peptic ulcer, and gastric cancer were selected for analysis. Frozen stocks within 50 subcultures were revitalized and grown on Brucella agar (Accumedia, USA) plates containing the same composition as Mueller-Hinton agar at 37°C under 10% CO_2_ and 100% humidity.

### Preparation of Genomic DNA from *H. pylori*

Genomic DNA was extracted from *H. pylori* as described previously [39]. Briefly, cultured bacterial colonies were collected and suspended in 1 ml of phosphate-buffered saline (PBS, pH 7.2). Bacterial cells were harvested by centrifugation at 12,700 ×*g* for 5 min and suspended in 200 μl of Tris-EDTA (TE) buffer (pH 7.0). After adding 600 μg of lysozyme (Genery, China), the cell mixture was incubated at 37°C for 1 h. Then, after adding 20 μl of 10%SDS solution and 10 μg of RNase (RBC, Taiwan), the cell mixture was incubated at 37°C for 1 h, followed by treatment with 120 μg of Protease K (GeNet Bio, Korea) and 172 μg of pronase (Merck) at 37°C for 1 h. After adding 1/10 volume of 5% cetyltrimethylammonium bromide (BDH Chemicals Ltd., England)-0.5 M NaCl solution and incubating at 37°C for 1 h, the mixture was chloroform extracted by adding an equal volume of phenol:chloroform:isoamyl alcohol (25:24:1) solution and vortexing. After centrifuging at 12,700 ×*g* for 10 min, the aqueous layer was collected, mixed with 1/10 volume of 3 M sodium acetate (pH 5.2), and added to an equal volume of isopropanol. After incubating at −70°C for 20 min, the sample was centrifuged at 12,700 ×*g* for 10 min. DNA pellets were washed with 1 mL of 70% ethanol, dried completely, dissolved in 30 μl of TE buffer, and frozen at -70°C until required.

### PCR

Extracted DNA (50 ng) was PCR amplified with AccuPower PCR PreMix using 10 pmol of forward and reverse primers listed in Table 1. After pre-denaturation at 94°C for 4 min, PCR was carried out at the annealing temperature for 1 min with extension at 72°C for 30 sec, followed by a final extension at 72°C for 7 min. The annealing temperatures and number of cycles are listed in Table 1. Amplified DNA was separated by 1% agarose gel electrophoresis, stained with ethidium bromide, and visualized and analyzed with a Fluor-S MultiImager (Bio-Rad, USA).

For nucleotide sequence analysis, PCR was performed using the *pfu* PCR Kit (Cat. No. EBT-11011, ELPIS, Korea), 4 ng of genomic DNA, 10 pM of primers, and 0.5 unit of *pfu* DNA polymerase under the conditions described above. Amplified PCR product was purified using an EZ-Pure PCR Purification Kit (Cat. No. EP201-50N, Enzynomics, Korea). Purified PCR products were sequenced using an Applied Biosystems 3730xl DNA Analyzer (Carlsbad, USA).

### Vacuolation Activity Test

Vacuolation activity was measured using RK-13 (ATCC CCL-37) cells as described previously with slight modifications [40]. Briefly, RK-13 cells were grown overnight in RPMI 1640 medium (Gibco) supplemented with 5% fetal bovine serum (FBS; Gibco), adjusted to 1.5 × 10^4^ cells/100 μl, added in 96-well microplates at 100 μl/well and incubated for 4 h. *H. pylori* strains were grown in a thin-layer liquid culture system [1]. Briefly, bacteria were grown overnight on a Petri dish (100 mm diameter) containing 3 ml of Brucella broth (Accumedia) supplemented with 10% bovine serum (Gibco) at 37°C for 24 h in an atmosphere of 10% CO_2_ and 100% humidity. Supernatants (50 μl) were harvested, serially (1:2) diluted with the culture medium, added into 96-well plates of RK-13 cells, and incubated at 37°C for 12 h. The degree of vacuolation was observed under a light microscope (Olympus, Japan). Vacuolation activity was defined as the reciprocal of the dilution factor of the culture supernatant in which 10% of cells were vacuolated. Vacuolation activity was defined as the reciprocal of the dilution factor of the culture supernatant in which 10% of cells were vacuolated.

### Statistical Analysis

The two-way association between genotypes of virulent factors and clinical entities of gastric disease was examined using the Chi-square test and Fisher's exact test. The distribution of patient ages among clinical entities of gastric disorder was assessed with an independent two-sample *t*-test. A *p*-value of < 0.05 was considered statistically significant. Odds ratios (ORs) adjusted for sex were given with 95% confidence intervals to estimate the risk. All data were statistically analyzed using the Statistical Package for Social Sciences 22 (SPSS Inc., USA).

## Results

### Patients and *H. pylori* Isolates

**Fig. 1:**
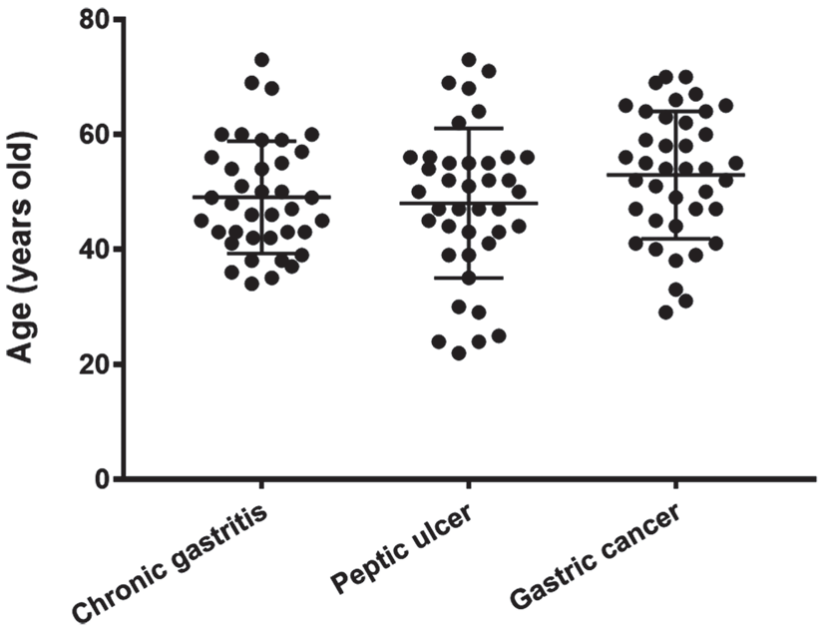
Age distribution of patients with chronic gastritis, peptic ulcer, or gastric cancer.

A total of 116 *H. pylori* strains were recovered from patients diagnosed clinically and pathologically with chronic gastritis (38 strains; male/female, 18/20), peptic ulcers (39 strains; male/female, 32/6), and gastric cancers (39 strains; male/female, 25/14) [35-37]. Patients were from the southern area of the Korean peninsula. The average patient age with standard deviation was 48.6 ± 12.1 years for those with chronic gastritis, 48.4 ± 13 years for those with peptic ulcer, and 52.9 ± 11.1 years for those with gastric cancer. Patient ages did not significantly differ from each other, although the average age of patients with gastric cancer was higher than that of others (Fig. 1). Strains that had been subcultured less than 50 times were chosen for this study to minimize the probability of genomic mutation during in vitro maintenance.

### Polymorphism of *vacA* Alleles

**Fig. 2:**
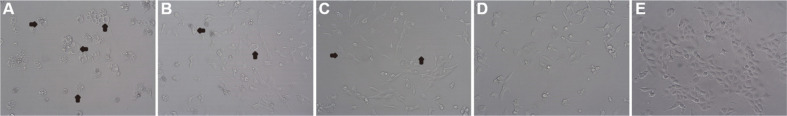
Vacuolating toxic effects of *H. pylori* culture broth on RK-13 cells. The RK-13 cells showed vacuolation after 12 h of coculture with a two-fold diluted culture medium of each *H. pylori* strain. Arrows indicate well-characterized vacuolation. A, extremely strong; B, strong; C, moderate; D, weak; E, non-toxic effect control.

*vacA* allele polymorphisms are determined by variation in the signal sequence region (*s1a*, *s1b*, *s1c*, and *s2*), mid-region (*m1* and *m2*), and intermediate region (*i1*, *i2*, and *i3*). In this study, the *vacA* genotype in the signal sequence region was the *s1* type for all 116 *H. pylori* strains, whereas 109 strains (94%) were grouped as the *m1* type in the mid-region, and 110 strains (95%) were grouped as the *i1* type in the intermediate region (Table 2). No strains exhibited the *s2* type. Only 6% and 2.5% of strains were grouped as *m2* and *i2* types, respectively. The *s1* subtypes *s1a* and *s1c* were identified in 37 (32%) and 79 (68%) strains, respectively, whereas the *s1b* subtype was not detected. Eighteen strains associated with chronic gastritis (47%) were grouped into the *s1a* subtype, which was more than the number of strains associated with gastric cancer (eight strains, 21%) grouped into this subtype. In contrast, 31 (79%) strains associated with gastric cancer were grouped into the *s1c* subtype, which was significantly (*p* = 0.017) higher than the number (20 strains, 53%) of strains associated with chronic gastritis grouped into this subtype. No significant differences were found when vacuolating toxin activity was measured, although the average value for the gastric cancer-associated strains was higher than that of those associated with chronic gastritis or peptic ulcer (Fig. 2 and Table 2). There was no significant difference in vacuolating toxin activity between the *s1a* and *s1c* subtypes. However, 33% of gastric cancer strains were classified into the extremely strong group, which was significantly (*p* = 0.026) higher than that of chronic gastritis strains or peptic ulcer strains when toxin activity was classified into the following four groups: extremely strong (> 41, more than the average value plus 1 SD); strong (41–21.8, from less than the extremely strong to the average); weak (21.7–2.6, from the average value to the average minus 1 SD); and extremely weak (< 2.6, less than the value of the average minus 1 SD) (Table 3).

### Genotypes of *cagA*

The *cagA* gene was found in almost all strains, with the exception of two strains isolated from patients with chronic gastritis. Polymorphisms in the 3'-region of *cagA* were analyzed by PCR and nucleotide sequencing. Five kinds of repeat patterns of EPIYA were identified in *cagA*-positive *H. pylori* isolates, and most *H. pylori* isolates grouped into the EPIYA-ABD pattern, which has been frequently isolated in Asia. Four patterns of variant EPIYA-ABDs were found in six strains by PCR and nucleotide sequencing (Table 4). There was no EPIYA motif in the EPIYA-B segment or deletion of amino acid residues in the downstream region of EPIYA-A and/or -D segments. Multiple EPIYA-AB motifs were found in one chronic gastritis strain and one gastric cancer strain. Multiple EPIYA-D segments (EPIYA-ABD′bD and A′bD′bD) were present in one peptic ulcer strain and in three gastric cancer strains.

### Genotypes of Adhesion Genes

The genotypes of the adhesion genes *alpA*, *alpB*, *babA1*, *babA2*, *hopZ*, *oipA*, and *sabA* were examined by PCR. Among the 116 strains, *alpA*, *alpB*, *babA2*, *hopZ*, *oipA*, and *sabA* were detected in 88 (76%), 113 (97%), 91 (79%), 96 (83%), 112 (97%), and 70 (60%) strains, respectively (Table 5). The prevalence of *alpA* in peptic ulcer strains (82%) and gastric cancer strains (87%) was significantly (*p* = 0.026 and 0.005, respectively) higher than that in chronic gastritis strains (58%), and the prevalence of *babA2* in peptic ulcer (95%) or gastric cancer strains (95%) was also significantly (*p* = 0.000) higher than that in chronic gastritis strains (45%). The prevalence of *hopZ* was significantly (*p* = 0.036) higher in the gastric cancer strain (92%) than that in the chronic gastritis strains (73%). The prevalence of *alpB*, *oipA*, and *sabA* did not differ significantly among the three clinical entities of gastric diseases (Table 5).

### Genotypes of Genes in the Endonuclease Restriction/Modification Systems

Genes in the endonuclease restriction/modification systems were used for genotyping *H. pylori* isolates, including *hrgA*, *hyp*III, and *iceA*. Among the 116 strains, *hrgA*, *hyp*IIIR, and *hyp*IIIM were detected in 49 (35%), 79 (69%), and 116 (100%) strains, respectively. The *iceA1* and *iceA2* genotypes were found in 98 (84%) and 15 (13%) strains, respectively. The prevalence of *hrgA* in chronic gastritis (44%) strains was higher than that in peptic ulcer (21%) or gastric cancer (33%), although there was no significant difference among the three groups. The prevalence of *hyp*III and *iceA* was not significantly associated with clinical outcomes (Table 6).

### Genotypes of *dupA* Genes in CagPAI

Genotyping of *dupA* genes was performed by PCR-based identification of *jhp0917* and *jhp0918*; *dupA* was detected in 28 (24%) strains while *jhp0918* was found in 2 (2%) strains. The prevalence of *dupA* in peptic ulcer strains (26%) and gastric cancer strains (28%) was higher than that in chronic gastritis strains (18%), although this lacked statistical significance (Table 7).

### Contribution of Genotyping Factors and Vacuolating Toxicity to the Development of Severe Gastric Symptoms

Among the bacterial factors analyzed in this study, the prevalence of genotypes *vacAs1c*, *alpA*, *babA2*, and *hopZ* and the extremely strong vacuolating toxicity showed significant differences between strains associated with chronic gastritis strains and those associated with severe symptoms (peptic ulcer and gastric cancer). The presence of the *babA2* genotype contributed the most to the development of peptic ulcer (OR: 27.5; 95% CI: 5.0–151.2; *p* = 0.000) and gastric cancer (OR: 21.7; 95% CI: 4.4–106.3; *p* = 0.000) (Table 8). The presence of *alpA* also contributed to the development of peptic ulcer (OR: 7.0; 95% CI: 2.0–24.9; *p* = 0.026) and gastric cancer (OR: 4.5; 95% CI: 1.2–17.6; *p* = 0.005). Genotypes of *vacAs1c* and *hopZ* also showed significant associations with the development of gastric cancer. Extremely strong vacuolating toxicity was significantly associated with the development of gastric cancer compared with that of chronic gastritis (OR: 3.5; 95% CI: 1.1–11.2; *p* = 0.009)(Table 8).

### Combination of Genotypes Predicting the Development of Peptic Ulcer and Gastric Cancer

A total of 11 combinations generated by double, triple, and quadruple genotypes of *vacAs1c*, *alpA*, *babA2*, and *hopZ* were significantly associated with the development of severe symptoms (Table 9). Among these combinations, *babA2*/*hopZ* was present at the highest proportion (82.1%) in severe symptom strains, followed by *alpA*/*babA2* (79.5%), *alpA*/*hopZ* (75.6%), *vacAs1c*/*babA2* (71.8%), and *alpA*/*babA2*/*hopZ* (70.5%). The combination of *vacAs1c*/*alpA*/*babA2* was the most predictable determinant for the development of severe symptoms (OR: 15.8; 95% CI: 4.6–53.8; *p* = 0.000), followed by *vacAs1c*/*alpA*/*babA2*/*hopZ* (OR: 11.6; 95% CI: 3.4–39.6; *p* = 0.000), *alpA*/*babA2* (OR: 10.9; 95% CI: 3.9–30.5; *p* = 0.000), *vacAs1c*/*babA2* (OR: 8.4; 95% CI: 3.0–23.6; *p* = 0.000), and *vacAs1c*/*babA2*/*hopZ* (OR: 8.6; 95% CI: 3.0–25.0; *p* = 0.000).

Among the four genotype markers, the OR average of double and triple combination groups containing *babA2* showed the highest value when compared with combinations containing other genotype markers, demonstrating that *babA2* might be the most critical determinant in the development of severe symptoms of gastric diseases (Tables 8 and 9).

## Discussion

Toxigenic strains of *H. pylori* carrying *cagA* and *vacA* are known to be closely associated with the development of gastric diseases such as peptic ulcer and gastric cancer [2, 10, 11, 14, 15]. Notably, although infections of *H. pylori* bearing *cagA* and *vacA* cause chronic gastritis in many cases, they are not always associated with peptic ulcer or gastric cancer [3]. Only approximately 10–15% of infected individuals were reported to develop peptic ulcer, gastric carcinoma, and MALT lymphoma [6, 19]. In Korea, most people are infected early in life with toxigenic *H. pylori* strains bearing *cagA* and *vacA* [20-22], but only a small proportion of these individuals have complained of severe gastric maladies [21-23]. These studies suggest that, along with *cagA* and *vacA*, other virulence factors of *H. pylori* are involved in disease outcomes.

A variety of candidate virulence factors have been identified for their role in provoking gastric pathogenesis. Unfortunately, their relevance to severe gastric disorders has proved epidemiologically controversial [3]. The reported discrepancies in the relationship between virulence factors and disease outcomes may be due to differences in prevalence rates and the types of *cagA* and *vacA* virulence factors present in different regions [3,5,26-28,41,42]. In contrast, most Korean *H. pylori* isolates contain *cagA* and *vacA*, thereby simplifying the analysis of the influence of other virulence factors and the understanding of their roles in the development of severe gastric symptoms. The risk prediction of severe gastric symptoms according to virulence factors is critical in the management and treatment of *H. pylori* infections. Therefore, in this study we investigated the relevance of virulence factors to clinical symptoms and sought to establish risk rates for the development of gastric diseases caused by *H. pylori* infections based on previously identified virulence factors.

Difference in vacuolating ability of *H. pylori* is conferred by *vacA* type variations in the signal sequence (*s1a*, *s1b*, *s1c*, and *s2*), mid-region (*m1* and *m2*), and intermediate region (*i1*, *i2*, and *i3*). Recently, a deletion-region (*d1* and *d2*) and c-region (*c1* and *c2*) have also been identified [3, 43]. Genotypes and subtypes of *vacA* in *H. pylori* strains have been reported to be geographically distributed. *H. pylori* strains in Northeast Asia have been predominantly grouped into the *s1*, *m1*, and *i1* types based on the *vacA* signal sequence, middle, and intermediate regions, respectively [16, 34]. Consistent with the results of previous studies [16, 34], most strains in this study were grouped as *s1/m1/i1*, irrespective of gastric symptoms, with a third of strains being classified as *s1a* subtype and two-thirds of strains being classified as *s1c* subtypes. Several studies have reported that subtypes *s1a* and *m1* are associated with peptic ulcer, intestinal metaplasia, or gastric cancer [15, 44, 45]. However, the proportion of *s1c* subtype was significantly higher in the gastric cancer group in this study (Table 2). The proportion of *s1c* was > 2.5-fold higher in peptic ulcer strains (72%) and > 3.5-fold higher in gastric cancer strains (79%) than those (28% in peptic ulcer strains and 21% in gastric cancer strains) of *s1a*, demonstrating that the *s1c* subtype may increase the risk of severe symptoms. Since all *H. pylori* strains carry different actual vacuolating abilities [3], the risk rates based on the expressed toxicity were analyzed. Values for the vacuolating cytotoxin activities of all 116 strains were widely distributed from 0 to 191 with no significant difference among gastric symptoms (Table 2). However, when vacuolating toxin activities were divided into four levels as shown in Table 3, gastric cancer strains were noticeably more classified into the extremely strong group than strains in other symptom groups (*p* < 0.05). This result might reflect the extent to which vacuolating toxin activity has a critical role in the development of gastric cancer.

The *vacAs1/i1/m1* gene is closely associated with *cagA* [3, 46], and therefore the repeat pattern of EPIYA of *cagA* in all 116 isolates was also investigated. Most East Asian *H. pylori* strains carry the *cagA* gene that encodes CagA proteins of the EPIYA-ABD type (Asian type CagA), irrespective of gastric symptoms [3, 21, 41]. Our results are consistent with these previous studies that demonstrated a low-level association of *cagA* with gastric cancer and peptic ulcer in Asian strains [6, 21]. In this study, since most isolates were found to be EPIYA-ABD type, the difference in risk rate between EPIYA type and multi-EPIYA type could not be identified.

The gastric mucus moves rapidly outward and *H. pylori* has to overcome the shedding flow of mucus by using bacterial factors to attach to the epithelium of the gastric mucosa. Therefore, *H. pylori* adherent factors play important roles in the initial colonization, long-term persistence, and gastric pathogenesis [47]. Several *H. pylori* adhesins belonging to the *H. pylori* outer membrane prion (HOP) subfamily, including BabA (HopS), SabA (HopP), AlpA (HopC), AlpB (HopB), HopZ, and OipA (HopH), have been extensively analyzed to understand bacterial interaction with the epithelial surface of the gastric mucosa [32].

In this study, the presence of *alpA*, *babA2*, and *hopZ* in *H. pylori* strains with severe gastric symptoms was significantly higher than that in chronic gastritis strains (Table 5). *alpA* is known as a virulence factor involved in signal transduction of host epithelial cells during *H. pylori* infection [4, 48]. Previous studies also demonstrated that an *H. pylori* *ΔalpA/B* mutant poorly colonized the stomachs of guinea pigs, mice, and Mongolian gerbils, indicating that AlpA and AlpB play an important role in bacterial colonization [24, 25, 48]. AlpA and AlpB have not yet been clearly identified as a virulence factor in humans and their role remains controversial [4]; however, in this study, we confirmed that the presence of *alpA* was significantly correlated with the development of gastric cancer in *H. pylori* infection (Table 5, *p* < 0.05).

Previous studies showed that the presence of the *babA2* gene substantially increased the risk of peptic ulcer development [28, 49]. In addition, a genome-wide association study on European *H. pylori* isolates demonstrated that, compared with isolates from gastritis patients, the gastric cancer phenotype was associated with the *babA2* gene [42]. Conversely, a meta-analysis study reported a lack of significant correlation in Asian populations [49]. This controversial result of the Asian type is probably due to the high overall prevalence of the *babA2* gene and significant heterogeneity in Asian isolates [3]. Limiting factors, such as the distinct genotypic profile of Western/Asian isolates and poor correlation between the presence of *babA2* gene and actual expression of BabA2 protein also hamper demonstrating the risk of the *babA2* gene on disease outcome [28, 50]. Although there is still controversy as to whether the *babA2* gene is related to the development of severe gastric symptoms, we observed the presence of the *babA2* gene with high frequency in patients with peptic ulcer and gastric cancer in this study (Table 5, *p* < 0.05).

*hopZ* was found to be regulated at the transcriptional level according to pH change [51], and *hopZ* expression depends on an on/off switch during early bacterial colonization [29]. These results indicate that *hopZ* has a strong selectivity in vivo, plays an important role in adaptation to the host environment [52], and has an essential role in colonization of the gastric mucosa during early infection [29]. Nevertheless, it is unclear whether *hopZ* is related to other virulence factors or is linked to other clinical diseases [29, 53]. However, we confirmed that the presence of the *hopZ* gene had a significant correlation with the disease outcome of gastric cancer (Table 5, *p* < 0.05).

Unlike *alpA*, *babA2*, and *hopZ*, the difference in gene prevalence of *alpB*, *opiA*, and *sabA* was difficult to determine according to the clinical outcomes. *alpB* has high homogeneity with *alpA* and is known to play a similar important role in adhering to gastric tissues and colonization [24, 25, 48]. In this study, most isolates from each clinical symptom group were found to have *alpB* (97%), and therefore *alpB* may not be a useful marker for predicting the clinical outcome of *H. pylori* infection. Previous studies reported that *oipA* is closely linked to the expression of *cagA* [31, 54], which was also confirmed in this study with only 2 out of 112 *oipA*(+) strains being *cagA*(-) strains. Yamaoka Y. et al. reported that *oipA* can be functionally turned “on” or “off ” by a slipped strand mispairing mechanism [6]. Several studies of prevalence and meta-analysis demonstrated that the *oipA* “on” function increased the risk of development of peptic ulcer and gastric cancer [55]. Conversely, an induced *oipA* “on” status is reported to have no relation to the risk of severe gastric symptoms [27, 54, 56]. This study focused on discovering virulence factors of *H. pylori* that could be detected by PCR and then used as markers of development of gastric symptoms, which would be more useful in clinical approaches than sequencing to identify the functional status of *oipA*. This limitation made it more difficult to find any link between *oipA* prevalence and disease outcome in this study. Moreover, *oipA* does not appear to be a useful marker for predicting the clinical outcome of *H. pylori* infection because most *H. pylori* isolates in Korea were identified as virulent strains [56].

*sabA* is known to be associated with chronic infection establishment of *H. pylori* [57]. The proportion of the *sabA* gene was slightly higher in chronic gastritis strains than in others, although this lacked statistical significance (Table 5). A survey in Japan reported that *sabA* was linked to gastric cancer [58]. However, although severe neutrophil infiltration and epithelial atrophy are associated with *sabA*, there are reports that *sabA* does not affect clinical outcome, and therefore the relationship between *sabA* and clinical diseases remains controversial [58, 59].

Alleles of restriction and modification systems, including *iceA* and *hrgA*, are reportedly predictive of gastric symptoms in East Asia [14, 33, 60]. However, the relationship between *H. pylori* *iceA*/*hrgA* and clinical outcomes is still controversial [5, 13, 61]. There are two main allelic variants, *iceA1* and *iceA2*, of which *iceA1* is reported to be predominant in East Asia [62]. Several studies have suggested that *iceA1* is frequently found in strains isolated from patients with peptic ulcer and gastric cancer [33, 60, 62], whereas others have shown different findings [16, 63], even though *iceA1* is presumed to facilitate neutrophil filtration and inflammation [62, 64]. In this study, the prevalence of *iceA1* was higher in peptic ulcer- and gastric cancer-associated strains in comparison with that of *iceA2*, and significant discrepancies were not found. *H. pylori* has a highly heterogeneous and variable type II R-M system, whereas the *hyp*III R-M system contains two genes, *hyp*IIIR and *hyp*IIIM [12]. The *hrgA* gene, which is considered an important virulence determinant in *H. pylori*-associated gastric diseases, can replace *hyp*IIIR [14]. Therefore, the correlation between *hrgA*/*hyp*IIIR status and clinical outcome has been used to determine *H. pylori* toxicity [14]. Subgroup analysis of possible correlations between clinical outcome and *hrgA*/*hpy*IIIR status suggested that the prevalence of the *hrgA* gene was increased among gastric cancer patients (42%) in East Asian countries compared with that in patients without gastric cancer (17%) [14]. In contrast, another study reported that *hrgA*/*hyp*IIIR status and *iceA* genotypes are not related to gastric outcomes, although regional differences in the prevalence between Asia and the West were observed [61]. In addition, these authors suggested that the prevalence of the *hrgA* gene was not related to other putative virulence factors, such as *cagA*, *vacA*, or *iceA*, in either East Asia or Western countries [61]. In this study, the prevalence of *hrgA* gene was higher, though not significantly, in chronic gastritis strains than in other strains, showing different results from the previous study. Thus, the results of studies on *hrgA* are contradictory. This may be because *hrgA* prevalence was not related to other putative virulence factors (*cagA*, *vacA*, or *iceA*), which play an important role in the development of pestilent gastric disease caused by *H. pylori* infection [22, 61].

Members of the pathogenic island region (CagPAI) of *H. pylori* containing the type IV secretion system have been proposed as playing a role in the pathogenesis of gastric diseases [12, 19]. *dupA* is a component gene of the type IV secretion system, which encompasses both *jhp0917* and *jhp0918*. *dupA* is the first identified disease-specific *H. pylori* virulence factor that induces duodenal ulcer and has a suppressive action on gastric cancer [22]. Despite the reported gastric cancer inhibitory function, a pooled analysis of data from three Western countries (the USA, Belgium, and South Africa) linked the presence of the *dupA* gene to peptic ulcer and gastric cancer [65]. In addition, although there is a large regional difference in prevalence of the *dupA* gene, this may be a risk factor for gastric cancer along with duodenal ulcer [66]. However, a meta-analysis using 11–12 studies on *dupA* (9 countries) reported that *dupA* had no correlation with the development of peptic ulcer and gastric cancer [67]. Other studies also reported no correlation between *dupA* and gastric disease outcome, and this association remains controversial [68, 69]. In this study, the prevalence of *dupA* (*jhp0917*) was higher, but not significantly, in peptic ulcer- and gastric cancer-associated strains than that in chronic gastritis-associated strains. The *dupA* gene may be mutated and protein expression may be inhibited. Therefore, a study based on DupA protein expression should be considered to accurately analyze the correlation between *dupA* expression and clinical outcome [6].

Prediction of the severe gastric disease outcome of *H. pylori* using one virulence factor is difficult. Many virulence factors have been correlated with each other. The *oipA* “on” status has been found to be associated with *cagA* and *vacA* [27, 30], and other major virulence factors have also been reported to be correlated [3]. Therefore, severe symptoms may originate from the complex results of several linked virulence factors, rather than the function of a single factor [3, 52, 53, 57]. Multiple correspondence analysis has been shown to serve as a better approach for the prediction of peptic ulcer, gastric cancer, and MALT lymphoma [26, 57]. Therefore, this study was conducted using the four identified virulence factors, *vacAs1c*, *alpA*, *babA2*, and *hopZ*, that showed significant discrepancies in their prevalence between chronic gastritis strains and severe symptoms, to investigate whether clinical outcome could be predicted with each combination. Eleven combinations of genotypes using the four virulence factors were generated (Table 9), all of which showed an OR value of 3.3 or higher (*p* < 0.001). These results confirmed that all four factors could affect gastric symptoms and be used as markers for disease outcome. Among the combinations, the triple genotype *vacAs1c*/*alpA*/*babA2* was the most predictable for the development of severe symptoms (OD: 15.8). The average OR of double and triple combination groups containing *babA2* was significantly higher than that of the other groups. Taken together, these data suggest that the genotype factor *babA2* might contribute more to the development of severe gastric symptoms than others.

Various other factors, as well as genotype factors, play an important role in the development of severe clinical symptoms, including age, sex, genetic characteristics, diet conditions, and underlying diseases [3, 54]. The link between gene presence and functional protein expression must also be considered [28]. In addition, the possibility of complex *H. pylori* infections in which different genotype combinations coexist cannot be ruled out [70]. In this case, only one strain of *H. pylori* was isolated from each patient, making it difficult to analyze the correlation between virulence factors and disease outcome. The large regional differences in the prevalence of virulence factors also impose limitations, causing bias in the analysis results. In this study, 116 strains of *H. pylori* isolates from patients with no significant age difference were analyzed (Fig. 1): all strains were *vacA*s1 genopositive while 114 strains were *cagA* genopositive. The OR value for the development of gastric symptoms and the statistical significance in the relevance of genotype factors to gastric symptoms were calculated by the adjustment of sex using the Mantel-Haenszel method [71] to overcome gender imbalance. However, other limiting factors in this study remain a challenge.

This study attempted to identify candidate virulence factors of *H. pylori* that can predict severe gastric symptoms via PCR screening. Since this study analyzed *H. pylori* isolated over a limited period of time, it could not provide information on the current prevalence of the virulence factors. However, we found that the presence of *vacAs1c*, *alpA*, *babA2*, and *hopZ* genes could increase the risk of disease outcome in infections with toxigenic *H. pylori* that also harbor the *cagA* and *vacA* genes. Moreover, we confirmed that severe gastric symptoms could be predicted at a high level of possibility through multiple correspondence analysis using a combination of these four genes. This could improve our understanding of the role of these virulence factors of *H. pylori*, which will assist in early prediction of disease outcome through the simple PCR method and thereby enable appropriate treatment.

## Figures and Tables

**Table 1:** Sequences of oligonucleotide primers and PCR conditions for the genotyping of *H. pylori* isolates.

Genes	primer F (5'⇒3')	Primer R (5‘⇒3’)	Annealing temp	Cycles	Amplified DNA size (bp)	References
*cagA*5’region	GATAACAGGCAAGCTTTTGATG	CTGCAAAAGATTGTTTGGCAGA	55°C	35	349	[16]
*cagA* EPIYA FR	ACCCTAGTCGGTAATGGG	GCAATTTTGTTAATCCGGTC	48°C	30	293~299	[72]
*cagA* EPIYA JSR	GCAATTTTGTTAATCCGGTC	GCTTTAGCTTCTGAYACYGC	48°C	30	222	[72]
*vacA s1/s2*	ATGGAAATACAACAAACACAC	CTGCTTGAATGCGCCAAAC	52°C	35	259/286	[73]
*vacA* *s1a* s1a	TCTYGCTTTAGTAGGAGC	CTGCTTGAATGCGCCAAAC	52°C	35	212	[16]
*vacA* *s1b* ss3	AGCGCCATACCGCAAGAG	CTGCTTGAATGCGCCAAAC	55°C	35	187	[73]
*vacA* *s1c* s1c	CTYCTTTAGTRGGGYTA	CTGCTTGAATGCGCCAAAC	55°C	35	213	[16]
*vacA* *m1/m2*	CAATCTGTCCAATCAAGCGAG	GCGTCTAAATAATTCCAAGG	52°C	35	570/645	[16]
*vacA* *i1*	TTAATTTAACGCTGTTTGAAG	GTTGGGATTGGGGGAATGCCG	53°C	35	426	[74]
*vacA* *i2*	GATCAACGCTCTGATTTGA	GTTGGGATTGGGGGAATGCCG	53°C	35	432	[74]
*alpA*	ACGCTTTCTCCCAATACC	AACACATTCCCCGCATTC	65°C	40	304	[75]
*alpB*	TCGCCGGGACTTTGGGCAAC	TGCGCTAAAGCGGCGTCCAA	55°C	35	505	Designed
*oipA*	CAAGCGCTTAACAGATAGGC	AAGGCGTTTTCTGCTGAAGC	55°C	35	450	[76]
*babA2*	AATCCAAAAAGGAGAAAAAACATGAAA	ATAGTTGTCTGAAAGATC	50°C	38	180	[26]
*hopZ*	GCCTGATATGGGTGGCATGGG	ATTTGATAGCCCGCGCTGAT	50°C	35	493	[77]
*sabA*	GAGCTATTGACCAGCTCAATG	TAGTTTGGATTCGTTCTCATTA	50°C	35	447	[78]
*iceA1*	TATTTCTGGAACTTGCGCAACCTGAT	GGCCTACAACCGCATGGATAT	55°C	30	642	[79]
*iceA2*	CGGCTGTAGGCACTAAAGCTA	TCAATCCTATGTGAAACAATGATCGTT	55°C	30	662	[79]
*iceA1*Δ94	GGTGAGTCGTTGGGTAAGCGTTACAGAATT	CACAACCATCATATTCAGCCTCCCCCTCATA	55°C	30	520	[79]
*hrgA*	TCTCGTGAAAGAGAATTTCC	TAAGTGTGGGTATATCAATC	55°C	30	682	[80]
*hpy*IIIR	CTCATTGCTGTGAGGGAT	TCTTGATAGGATCTTGCG	55°C	30	443	[80]
*hpy*IIIM	CTCATTGCTGTGAGGGAT	TCTTGATAGGATCTTGCG	55°C	30	562	[80]
*dupA jhp0917*	TGGTTTCTACTGACAGAGCGC	AACACGCTGACAGGACAATCTCCC	57°C	30	307	[22]
*dupA jhp0918*	CCTATATCGCTAACGCGCGCTC	AAGCTGAAGCGTTTGTAACG	57°C	30	276	[22]

**Table 2:** *vacA* genotypes and vacuolating toxin activity of *H. pylori* isolates recovered from patients with chronic gastritis, peptic ulcer, or gastric cancer.

*vacA* genotypes and Vac activity	Total (*n*=116)	Chronic gastritis (*n*=38)	Peptic ulcer (*n*=39)	Gastric cancer (*n*=39)
*s1*	116(100%)	38(100%)	39(100%)	39(100%)
*s1a*	37 (32%)	18 (47%)	11 (28%)	8 (21%)
*s1b*	0	0	0	0
*s1c*	79 (68%)	20 (53%)	28 (72%)^a^	31 (79%) ^b^
*s2*	0	0	0	0
*m1*	109 (94%)	36 (95%)	37 (95%)	36 (92%)
*m2*	7 (6%)	2 (5%)	2 (5%)	3 (8%)
*i1*	110 (95%)	35 (89%)	38 (97%)	37 (95%)
*i2*	3 (2.5%)	1 (3%)	0	2 (5%)
*i1*,*i2*(-)	3 (2.5%)	2 (5%)	1 (3%)	0
Vacuolating toxin activity	21.8±19.2	20.3±17.2	18.7±18.0	26.3±21.2

^a^*p* = 0.103 and ^b^*p* = 0.017 compared with that in chronic gastritis with Fisher’s exact test.

**Table 3:** Vacuolating toxin activity of *H. pylori* isolates recovered from patients with chronic gastritis, peptic ulcer, or gastric cancer.

Symptoms	No	Vacuolating toxin activity			

		Extremely Strong (>41)	Strong (41~21.8)	Moderate (21.8~2.6)	Weak (<2.6)
Chronic gastritis	38	5 (13%)	8 (21%)	20 (53%)	5 (13%)
Peptic ulcer	39	4 (10%)	7 (18%)	19 (49%)	9 (23%)
Gastric cancer	39	13 (33%)^a^	6 (15%)	17 (44%)	3 (8%)
Total	116	22 (19%)	21 (18%)	56 (48%)	17 (15%)

Parentheses indicate percentage of each group classified by vacuolating cytotoxin activity.

^a^*p* = 0.026 compared with groups of chronic gastritis or peptic ulcer using Fisher’s exact test.

**Table 4:** Repeat patterns of EPIYA in the 3'-region of *cagA* of *H. pylori* isolates recovered from patients with chronic gastritis, peptic ulcer, or gastric cancer.

CagA EPIYA types ^a^	Chronic gastritis (*n*=38)	Peptic ulcer (*n*=39)	Gastric cancer (*n*=39)
ABD or A′bD	35 (92.0%)	38 (97.4%)	35 (89.7%)
ABD′bD or A′bD′bD	0	1 (2.6%)	3 (7.7%)
ABABD	1 (2.7%)	0	1 (2.6%)
CagA negative	2 (5.3%)	0	0

^a^, A, B, and D indicate typical EPIYA segment, b notes the EPIYA-B segment deleted in the 5’-end region including EPIYA motif, and A′ and D′ indicate the segment deleted in the downstream region of EPIYA motif. ABD segments produced DNA fragments of 293~299 bp with *cagA* EPIYA FR primers and 222 bp with EPIYA JSR primers. When amplified DNAs of different sizes were produced, amplified DNAs were subjected to nucleotide sequence analysis.

**Table 5:** Genotypes of adhesion proteins of *H. pylori* isolates recovered from patients with chronic gastritis, peptic ulcer, or gastric cancer.

Genotypes	Total (*n*=116)	Chronic gastritis (*n*=38)	Peptic ulcer (*n*=39)	Gastric cancer (*n*=39)
*alpA*	88 (76%)	22 (58%)	32 (82%) ^a^	34 (87%) ^b^
*alpB*	113 (97%)	35 (92%)	39 (100%)	39 (100%)
*babA2*	91 (79%)	17 (45%)	37 (95%) ^c^	37 (95%) ^d^
*hopZ*	96 (83%)	28 (73%)	32 (82%)	36 (92%)^e^
*oipA*	112 (97%)	35 (92%)	38 (97%)	39 (100%)
*sabA*	70 (60%)	25 (66%)	23 (59%)	22 (56%)

^a^*p* = 0.026, ^b^*p* = 0.005, ^c^*p* = 0.000, ^d^*p* = 0.000, and ^e^*p* = 0.036 compared with those with chronic gastritis using Fisher’s exact test.

**Table 6:** Genotypes of endonuclease enzyme genes of *H. pylori* isolates recovered from patients with chronic gastritis, peptic ulcer, or gastric cancer.

Genes	Total (*n* = 116)	Chronic gastritis (*n* = 38)	Peptic ulcer (*n* = 39)	Gastric cancer (*n* = 39)
*hrgA*	36 (35%)	16 (44%)	8 (21%)	12 (30%)
*hyp*ⅢR	80 (70%)	22 (56%)	30 (77%)	27 (70%)
*hyp*ⅢM	116 (100%)	38 (100%)	39 (100%)	39 (100%)
iceA1	98 (84%)	30 (78%)	34 (87%)	33 (85%)
*iceA1Δ*94	2 (2%)	1 (3%)	0	1 (3%)
*iceA2*	15 (13%)	6 (16%)	4 (10%)	5 (13%)

**Table 7:** Genotypes of *dupA* in CagPAI of *H. pylori* isolates recovered from patients with chronic gastritis, peptic ulcer, or gastric cancer.

Genes	Total (*n* = 116)	Chronic gastritis (*n* = 38)	Peptic ulcer (*n* = 39)	Gastric cancer (*n* = 39)
*jhp0918*	2 (2%)	2 (5%)	0	0
*jhp0917*–*0918*	28 (25%)	7 (18%)	10 (26%)	11 (28%)

**Table 8:** Analysis of the contributory role of genotyping factors and vacuolating toxicity in the development of gastric cancer and peptic ulcer.

	*vacAs1c*		*alpA*		*babA2*		*hopZ*		Extremely strong vacuolating toxin	
				
	Peptic ulcer	Gastric cancer	Peptic ulcer	Gastric cancer	Peptic ulcer	Gastric cancer	Peptic ulcer	Gastric cancer	Peptic ulcer	Gastric cancer
OR	1.7	3.2	7.0	4.5	27.5	21.7	2.4	3.4	1.4	3.5
95% CI	0.60–5.2	1.1–9.8	2.0–24.9	1.2–17.6	5.0–151.2	4.4–106.3	0.7–8.8	0.6–18.2	0.3–6.0	1.1–11.2
*p* value	0.103	0.017	0.026	0.005	0.000	0.000	0.421	0.036	0.702	0.009

**Table 9:** The multiple correspondence analysis of the genotypes *vacAs1c*, *alpA*, *babA2*, and *hopZ* with gastric symptoms.

Combinations of genotypes											

Genotypes	Double						Triple				Quadruple
			
*vacAs1c*	+	+	+				+	+	+		+
*alpA*	+			+	+		+		+	+	+
*babA2*		+		+		+	+	+		+	+
*hopZ*			+		+	+		+	+	+	+
	
No. (%) in 38 chronic gastritis strains	10 (26.3)	8 (21.1)	15 (39.5)	11 (29.0)	18 (47.4)	13 (34.2)	4 (10.5)	6 (15.8)	8 (21.1)	10 (26.3)	4 (10.5)
	
No. (%) in of 78 severe symptoms strains	52 (66.7)	56 (71.8)	52 (66.7)	62 (79.5)	59 (75.6)	64 (82.1)	49 (62.8)	49 (62.8)	46 (59.0)	55 (70.5)	43 (55.1)
	
Odds ratio	7.0	8.4	3.3	10.9	4.0	8.9	15.8	8.6	6.5	7.4	11.6
95% CI	2.7–18.01	3.0–23.6	1.4–8.0	3.9–30.5	1.5–10.6	3.4–23.2	4.6–53.8	3.0–25.0	2.4–17.1	2.8–19.3	3.4–39.6
*p* value	0.000	0.000	0.008	0.000	0.002	0.000	0.000	0.000	0.000	0.000	0.000

## References

[1] Joo JS, Park KC, Song JY, Kim DH, Lee KJ, Kwon YC (2010). A thin‐layer liquid culture technique for the growth of *Helicobacter pylori*. Helicobacter.

[2] Blaser MJ, Atherton JC (2004). *Helicobacter pylori* persistence: biology and disease. J. Clin. Investig..

[3] Šterbenc A, Jarc E, Poljak M, Homan M (2019). *Helicobacter pylori* virulence genes. World J. Gastroenterol..

[4] Matsuo Y, Kido Y, Yamaoka Y (2017). *Helicobacter pylori* outer membrane protein-related pathogenesis. Toxins (Basel).

[5] Yamaoka Y, Graham DY (2014). *Helicobacter pylori* virulence and cancer pathogenesis. Future Oncol..

[6] Yamaoka Y (2010). Mechanisms of disease: *Helicobacter pylori* virulence factors. Nat. Rev. Gastroenterol. Hepatol..

[7] Malaty HM (2007). Epidemiology of *Helicobacter pylori* infection. Best Pract. Res. Clin. Gastroenterol..

[8] Youn HS, Baik SC, Cho YK, Woo HO, Ahn YO, Kim K (1998). Comparison of *Helicobacter pylori* infection between Fukuoka, Japan and Chinju, Korea. Helicobacter.

[9] Shin A, Kim J, Park S (2011). Gastric cancer epidemiology in Korea. J. Gastric Cancer.

[10] Ferlay J, Shin HR, Bray F, Forman D, Mathers C, Parkin DM (2010). Estimates of worldwide burden of cancer in 2008: GLOBOCAN 2008. Int. J. Cancer.

[11] Fock KM, Ang TL (2010). Epidemiology of *Helicobacter pylori* infection and gastric cancer in Asia. J. Gastroenterol. Hepatol..

[12] Alm RA (1999). Analysis of the genetic diversity of *Helicobacter pylori*: the tale of two genomes. J. Mol. Med..

[13] Shiota S, Suzuki R, Yamaoka Y (2013). The significance of virulence factors in *Helicobacter pylori*. J. Dig. Dis..

[14] Ando T, Wassenaar TM, Peek RM, Aras RA, Tschumi AI, van Doorn LJ (2002). A *Helicobacter pylori* restriction endonucleasereplacing gene, *hrgA*, is associated with gastric cancer in Asian strains. Cancer Res..

[15] Choe YH, Kim PS, Lee DH, Kim HK, Kim YS, Shin YW (2002). Diverse *vacA* allelic types of *Helicobacter pylori* in Korea and clinical correlation. Yonsei Med. J..

[16] Yamaoka Y, Kodama T, Gutierrez O, Kim JG, Kashima K, Graham DY (1999). Relationship between *Helicobacter pylori*
*iceA*, *cagA*, and *vacA* status and clinical outcome: studies in four different countries. J. Clin. Microbiol..

[17] Kao CY, Sheu BS, Wu JJ (2016). *Helicobacter pylori* infection: An overview of bacterial virulence factors and pathogenesis. Biomed. J..

[18] McClain MS, Beckett AC, Cover TL (2017). *Helicobacter pylori* vacuolating toxin and gastric cancer. Toxins (Basel).

[19] Kalali B, Mejías Luque R, Javaheri A, Gerhard M (2014). *H. pylori* virulence factors: influence on immune system and pathology. Mediators Inflamm.

[20] Rhee KH (1990). Prevalence of *Helicobacter pylori* infection in Korea. J. Korean Soc. Microbiol..

[21] Ko JS, Kim KM, Oh YL, Seo JK (2008). *cagA*, *vacA*, and *iceA* genotypes of *Helicobacter pylori* in Korean children. Pediatr. Int..

[22] Lu H, Hsu PI, Graham DY, Yamaoka Y (2005). Duodenal ulcer promoting gene of *Helicobacter pylori*. Gastroenterology.

[23] Kim N, Park RY, Cho SI, Lim SH, Lee KH, Lee W (2008). *Helicobacter pylori* infection and development of gastric cancer in Korea: long-term follow-up. J. Clin. Gastroenterol..

[24] Senkovich OA, Yin J, Ekshyyan V, Conant C, Traylor J, Adegboyega P (2011). *Helicobacter pylori* AlpA and AlpB bind host laminin and influence gastric inflammation in gerbils. Infect. Immun..

[25] De Jonge R, Durrani Z, Rijpkema SG, Kuipers EJ, van Vliet AH, Kusters JG (2004). Role of the *Helicobacter pylori* outer-membrane proteins AlpA and AlpB in colonization of the guinea pig stomach. J. Med. Microbiol..

[26] Gerhard M, Lehn N, Neumayer N, Borén T, Rad R, Schepp W (1999). Clinical relevance of the *Helicobacter pylori* gene for bloodgroup antigen-binding adhesin. Proc. Natl. Acad. Sci. USA.

[27] Zambon C, Navaglia F, Basso D, Rugge M, Plebani M (2003). *Helicobacter pylori*
*babA2*, *cagA*, and *s1*
*vacA* genes work synergistically in causing intestinal metaplasia. J. Clin. Pathol..

[28] Fujimoto S, Ojo OO, Arnqvist A, Wu JY, Odenbreit S, Haas R (2007). *Helicobacter pylori* BabA expression, gastric mucosal injury, and clinical outcome. Clin. Gastroenterol. Hepatol..

[29] Kennemann L, Brenneke B, Andres S, Engstrand L, Meyer TF, Aebischer T (2012). *In vivo* sequence variation in HopZ, a phasevariable outer membrane protein of *Helicobacter pylori*. Infect. Immun..

[30] Dossumbekova A, Prinz C, Mages J, Lang R, Kusters JG, Van Vliet AH (2006). *Helicobacter pylori* HopH (OipA) and bacterial pathogenicity: genetic and functional genomic analysis of *hopH* gene polymorphisms. J. Infect. Dis..

[31] Horridge DN, Begley AA, Kim J, Aravindan N, Fan K, Forsyth MH (2017). Outer inflammatory protein a (OipA) of *Helicobacter pylori* is regulated by host cell contact and mediates CagA translocation and interleukin-8 response only in the presence of a functional *cag* pathogenicity island type IV secretion system. Pathog. Dis..

[32] Odenbreit S (2005). Adherence properties of *Helicobacter pylori*: impact on pathogenesis and adaptation to the host. Int. J. Med. Microbiol..

[33] Peek Jr RM, Thompson SA, Donahue JP, Tham KT, Atherton JC, Blaser MJ (1998). Adherence to gastric epithelial cells induces expression of a *Helicobacter pylori* gene, *iceA*, that is associated with clinical outcome. Proc. Assoc. Am. Physicians.

[34] Kim SY, Woo CW, Lee YM, Son BR, Kim JW, Chae HB (2001). Genotyping *CagA, VacA* subtype, *IceA1*, and *BabA* of *Helicobacter pylori* isolates from Korean patients, and their association with gastroduodenal diseases. J. Korean Med. Sci..

[35] Baik SC, Kim JB, Cho MJ, Kim YC, Park CK, Ryou HH (1990). Prevalence of *Helicobacter pylori* infection among normal Korean adults. J. Korean Soc. Microbiol..

[36] Baik SC, Youn HS, Chung MH, Lee WK, Cho MJ, Ko GH (1996). Increased oxidative DNA damage in *Helicobacter pylori*infected human gastric mucosa. Cancer Res..

[37] Song GY, Chang MW (1999). Antibiotic susceptibility of *Helicobacter pylori* and the combination effect of antibiotics on the antibioticresistant *H. pylori* strains. J. Korean Soc. Microbiol..

[38] Monstein HJ, Nikpour Badr S, Jonasson J (2001). Rapid molecular identification and subtyping of *Helicobacter pylori* by pyrosequencing of the 16S rDNA variable V1 and V3 regions. FEMS Microbiol. Lett..

[39] Taylor NS, Fox JG, Akopyants NS, Berg DE, Thompson N, Shames B (1995). Long-term colonization with single and multiple strains of *Helicobacter pylori* assessed by DNA fingerprinting. J. Clin. Microbiol..

[40] Ohta-Tada U, Takagi A, Koga Y, Kamiya S, Miwa T (1997). Flagellin gene diversity among *Helicobacter pylori* strains and IL-8 secretion from gastric epithelial cells. Scand. J. Gastroenterol..

[41] Park JY, Forman D, Waskito LA, Yamaoka Y, Crabtree JE (2018). Epidemiology of *Helicobacter pylori* and CagA-positive infections and global variations in gastric cancer. Toxins (Basel).

[42] Berthenet E, Yahara K, Thorell K, Pascoe B, Meric G, Mikhail JM (2018). A GWAS on *Helicobacter pylori* strains points to genetic variants associated with gastric cancer risk. BMC Biol..

[43] Soyfoo DM, Doomah YH, Xu D, Zhang C, Sang HM, Liu YY (2021). New genotypes of *Helicobacter pylori* VacA d-region identified from global strains. BMC Mol. Cell. Biol..

[44] Aydin F, Kaklikkaya N, Ozgur O, Cubukcu K, Kilic A, Tosun I (2004). Distribution of *vacA* alleles and *cagA* status of *Helicobacter pylori* in peptic ulcer disease and non‐ulcer dyspepsia. Clin. Microbiol. Infect..

[45] Höcker M, Hohenberger P (2003). *Helicobacter pylori* virulence factors-one part of a big picture. Lancet.

[46] Homan M, Luzar B, Kocjan BJ, Mocilnik T, Shrestha M, Kveder M (2009). Prevalence and clinical relevance of *cagA*, *vacA*, and *iceA* genotypes of *Helicobacter pylori* isolated from Slovenian children. J. Pediatr. Gastroenterol. Nutr..

[47] Posselt G, Backert S, Wessler S (2013). The functional interplay of *Helicobacter pylori* factors with gastric epithelial cells induces a multi-step process in pathogenesis. Cell Commun. Signal..

[48] Lu H, Wu JY, Beswick EJ, Ohno T, Odenbreit S, Haas R (2007). Functional and intracellular signaling differences associated with the *Helicobacter pylori* AlpAB adhesin from Western and East Asian strains. J. Biol. Chem..

[49] Chen MY, He CY, Meng X, Yuan Y (2013). Association of *Helicobacter pylori*
*babA2* with peptic ulcer disease and gastric cancer. World J. Gastroenterol..

[50] Homan M, Šterbenc A, Kocjan BJ, Luzar B, Zidar N, Poljak M (2014). Prevalence of the *Helicobacter pylori*
*babA2* gene and correlation with the degree of gastritis in infected Slovenian children. Antonie Van Leeuwenhoek..

[51] Merrell DS, Goodrich ML, Otto G, Tompkins LS, Falkow S (2003). pH-regulated gene expression of the gastric pathogen *Helicobacter pylori*. Infect. Immun..

[52] Xu C, Soyfoo DM, Wu Y, Xu S (2020). Virulence of *Helicobacter pylori* outer membrane proteins: An updated review. Eur. J. Clin. Microbiol. Infect. Dis..

[53] Servetas SL, Kim A, Su H, Cha JH, Merrell DS (2018). Comparative analysis of the Hom family of outer membrane proteins in isolates from two geographically distinct regions: the United States and South Korea. Helicobacter.

[54] Farzi N, Yadegar A, Aghdaei HA, Yamaoka Y, Zali MR (2018). Genetic diversity and functional analysis of *oipA* gene in association with other virulence factors among *Helicobacter pylori* isolates from Iranian patients with different gastric diseases. Infect. Genet. Evol..

[55] Sallas ML, Dos Santos MP, Orcini WA, David ÉB, Peruquetti RL, Payão SLM (2019). Status (on/off) of *oipA* gene: their associations with gastritis and gastric cancer and geographic origins. Arch. Microbiol..

[56] Torres K, Valderrama E, Sayegh M, Ramírez JL, Chiurillo MA (2014). Study of the *oipA* genetic diversity and EPIYA motif patterns in *cagA*-positive *Helicobacter pylori* strains from Venezuelan patients with chronic gastritis. Microb. Pathog..

[57] Lehours P, Ménard A, Dupouy S, Bergey B, Richy F, Zerbib F (2004). Evaluation of the association of nine *Helicobacter pylori* virulence factors with strains involved in low-grade gastric mucosa-associated lymphoid tissue lymphoma. Infect. Immun..

[58] Yamaoka Y, Ojo O, Fujimoto S, Odenbreit S, Haas R, Gutierrez O (2006). *Helicobacter pylori* outer membrane proteins and gastroduodenal disease. Gut.

[59] Yanai A, Maeda S, Hikiba Y, Shibata W, Ohmae T, Hirata Y (2007). Clinical relevance of *Helicobacter pylori*
*sabA* genotype in Japanese clinical isolates. J. Gastroenterol. Hepatol..

[60] Kidd M, Peek R, Lastovica A, Israel D, Kummer A, Louw J (2001). Analysis of *iceA* genotypes in South African *Helicobacter pylori* strains and relationship to clinically significant disease. Gut.

[61] Lu H, Graham DY, Yamaoka Y (2004). The *Helicobacter pylori* restriction endonuclease-replacing gene, *hrgA*, and clinical outcome: comparison of East Asia and Western countries. Dig. Dis. Sci..

[62] Yakoob J, Abbas Z, Khan R, Salim SA, Abrar A, Awan S (2015). *Helicobacter pylori*: correlation of the virulence marker *iceA* allele with clinical outcome in a high prevalence area. Br. J. Biomed. Sci..

[63] Ashour AAR, Collares GB, Mendes EN, de Gusmão VrR, de Magalhães Queiroz DM, Magalhães PP (2001). *iceA* genotypes of *Helicobacter pylori* strains isolated from Brazilian children and adults. J. Clin. Microbiol..

[64] Sgouras DN, Trang TTH, Yamaoka Y (2015). Pathogenesis of *Helicobacter pylori* infection. Helicobacter.

[65] Argent RH, Burette A, Miendje Deyi VY, Atherton JC (2007). The presence of *dupA* in *Helicobacter pylori* is not significantly associated with duodenal ulceration in Belgium, South Africa, China, or North America. Clin. Infect. Dis..

[66] Hussein N (2010). The association of *dupA* and *Helicobacter pylori*-related gastroduodenal diseases. Eur. J. Clin. Microbiol. Infect. Dis..

[67] Shiota S, Matsunari O, Watada M, Hanada K, Yamaoka Y (2010). Systematic review and meta-analysis: the relationship between the *Helicobacter pylori*
*dupA* gene and clinical outcomes. Gut Pathog..

[68] Pacheco A, Proença-Módena J, Sales A, Fukuhara Y, Da Silveira W, Pimenta-Módena J (2008). Involvement of the *Helicobacter pylori* plasticity region and cag pathogenicity island genes in the development of gastroduodenal diseases. Eur. J. Clin. Microbiol. Infect. Dis..

[69] Nguyen L, Uchida T, Tsukamoto Y, Kuroda A, Okimoto T, Kodama M (2010). *Helicobacter pylori*
*dupA* gene is not associated with clinical outcomes in the Japanese population. Clin. Microbiol. Infect..

[70] Kim JW, Kim JG, Chae SL, Cha YJ, Park SM (2004). High prevalence of multiple strain colonization of *Helicobacter pylori* in Korean patients: DNA diversity among clinical isolates from the gastric corpus, antrum and duodenum. Korean. J. Intern. Med..

[71] dos Santos Silva I (1999). Cancer epidemiology: principles and methods.

[72] Yamaoka Y, El-Zimaity HMT, Gutierrez O, Figura N, Kim JK, Kodama T (1999). Relationship between the *cagA*3 repeat region of *Helicobacter pylori*, gastric histology, and susceptibility to low pH. Gastroenterology.

[73] Atherton JC, Cao P, Peek RM, Tummuru MKR, Blaser MJ, Cover TL (1995). Mosaicism in vacuolating cytotoxin alleles of *Helicobacter pylori*: association of specific *vacA* types with cytotoxin production and peptic ulceration. J. Biol. Chem..

[74] Rhead JL, Letley DP, Mohammadi M, Hussein N, Mohagheghi MA, Hosseini ME (2007). A new *Helicobacter pylori* vacuolating cytotoxin determinant, the intermediate region, is associated with gastric cancer. Gastroenterology.

[75] Rokbi B, Seguin D, Guy B, Mazarin V, Vidor E, Mion F (2001). Assessment of *Helicobacter pylori* gene expression within mouse and human gastric mucosae by real-time reverse transcriptase PCR. Infect. Immun..

[76] Yamaoka Y, Kwon DH, Graham DY (2000). A *M_r_* 34,000 proinflammatory outer membrane protein (*oipA*) of *Helicobacter pylori*. Proc. Natl. Acad. Sci. USA.

[77] Peck B, Ortkamp M, Diehl KD, Hundt E, Knapp B (1999). Conservation, localization and expression of HopZ, a protein involved in adhesion of *Helicobacter pylori*. Nucleic Acids Res..

[78] De Jonge R, Pot RGJ, Loffeld RJLF, Van Vliet AHM, Kuipers EJ, Kusters JG (2004). The functional status of the *Helicobacter pylori*
*sabB* adhesin gene as a putative marker for disease outcome. Helicobacter.

[79] Mukhopadhyay AK, Kersulyte D, Jeong JY, Datta S, Ito Y, Chowdhury A (2000). Distinctiveness of genotypes of *Helicobacter pylori* in Calcutta, India. J. Bacteriol..

[80] Ando T, Wassenaar TM, Peek RM, Aras RA, Tschumi AI, van Doorn L-J (2002). A *Helicobacter pylori* restriction endonucleasereplacing gene, *hrgA*, is associated with gastric cancer in Asian strains. Cancer Res..

